# Stent implantation for a totally occluded right coronary artery in a six-year-old boy after Kawasaki disease: a case report

**DOI:** 10.1186/1752-1947-6-111

**Published:** 2012-04-16

**Authors:** Ya-Chi Hsu, Kae-Woei Liang, Ming-Chih Lin, Yun-Ching Fu, Sheng-Ling Jan

**Affiliations:** 1 Department of Pediatrics, Taichung Veterans General Hospital, Taichung, Taiwan; 2 Department of Pediatrics, National Yang-Ming University, Taipei, Taiwan; 3 Graduate Institute of Epidemiology and Preventive Medicine, National Taiwan University, Taipei, Taiwan; 4 Cardiovascular Center, Taichung Veterans General Hospital, Taichung, Taiwan

## Abstract

**Introduction:**

Coronary stenting has previously been considered to be less feasible in children under 12 years old due to the limitation of vascular access. We report the case of a six-year-old boy who successfully underwent stent implantation for his totally occluded right coronary artery.

**Case presentation:**

A Taiwanese boy aged six years and nine months old was found to have giant aneurysms after an acute episode of Kawasaki disease. An angiography revealed that his middle right coronary artery was totally occluded. A 0.014-inch guidewire was advanced to cross the totally occluded site. After pre-dilating the middle portion of his right coronary artery with a 1.5 mm balloon, stenting of his right coronary artery was accomplished using a 2.5 × 28 mm and a 2.5 × 18 mm bare metal stent. A final angiography demonstrated no residual stenosis or dissection.

**Conclusion:**

Coronary stenting could be a therapeutic option for children as young as six years old. Close follow-up is mandatory because the long-term outcome is still unclear, especially in a small child.

## Introduction

Kawasaki disease is characterized by systemic vasculitis. Its long-term morbidity and mortality are mainly related to the coronary artery sequelae [1]. Coronary stenting has previously been considered to be less feasible for children under 12 years old due to limitations of vascular access [2]. Here, we report the case of a six-year-old boy who successfully underwent stent implantation for his totally occluded right coronary artery. The success of this case suggests that coronary stenting could be a therapeutic option for patients as young as six years old.

## Case presentation

A Taiwanese boy aged six years and nine months old, with a history of an episode of incomplete Kawasaki disease at the age of five years and three months, was found to have complications in the form of giant aneurysms in both his right and left anterior descending coronary arteries [3]. He was followed-up and received regular aspirin and warfarin. During this period, electrocardiography revealed no evidence of ST-T changes. The boy did not experience any episode of angina or exercise intolerance. Multidetector computed tomography was arranged one year after the acute episode. It revealed thrombus formation and critical stenosis over his right coronary artery. Cardiac catheterization was therefore arranged. Our patient was 111 cm tall and weighed 20.2 kg. His body surface area was 0.80 m^2^.

**Figure 1:**
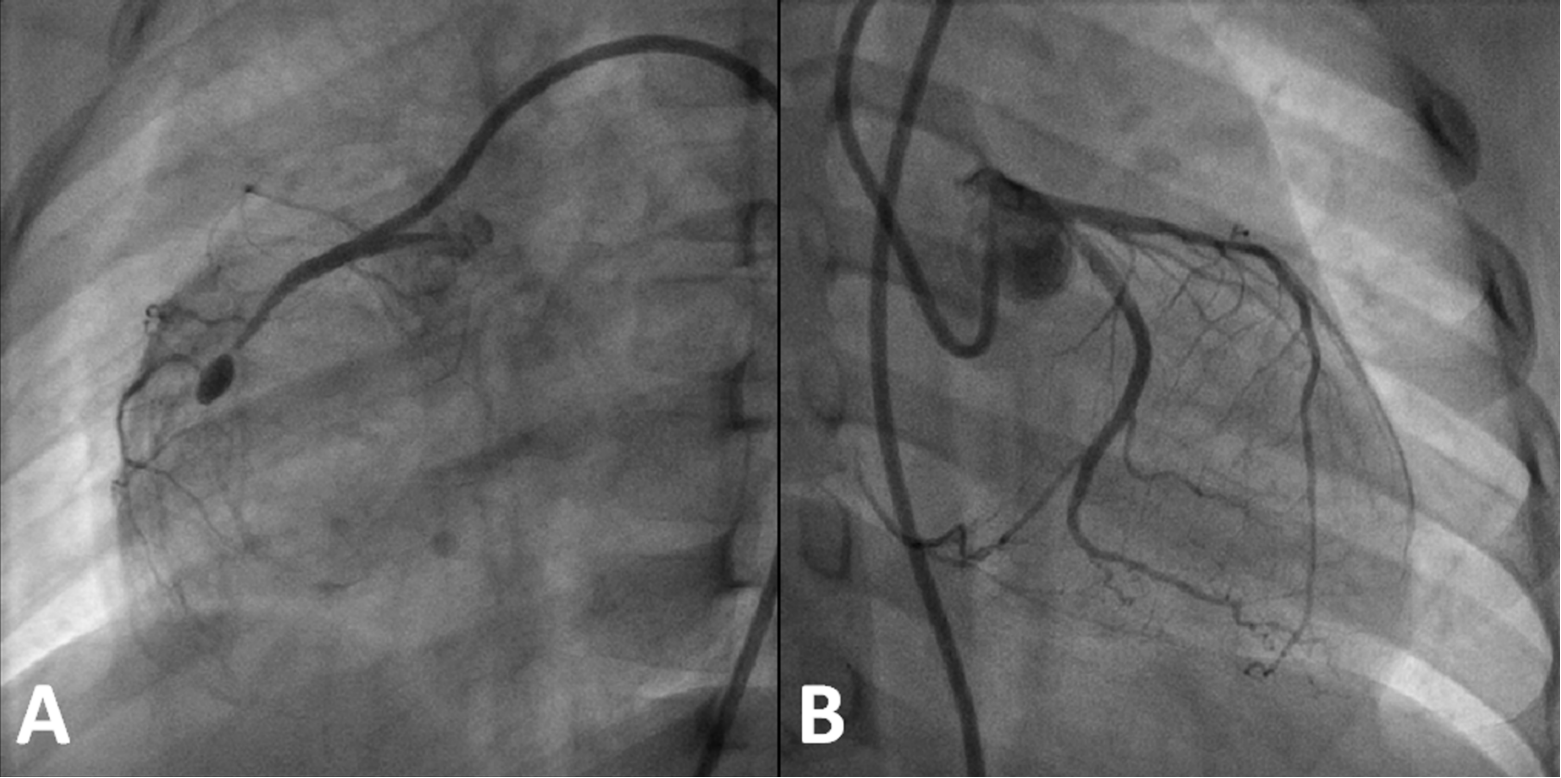
**Images from the initial angiography**. **(A) **Right coronary angiography in 18 degree right anterior oblique projection showing the totally occluded middle right coronary artery with a dilated aneurysm. **(B) **Left coronary angiography in 25 degree right anterior oblique and 9 degree caudal projection demonstrating the patent left main coronary artery and left circumflex coronary artery with 30% stenosis over the middle left anterior descending coronary artery.

Angiography revealed patent left main and left circumflex coronary arteries, with 30% stenosis over his middle left anterior descending coronary artery and a totally occluded middle right coronary artery with a dilated aneurysm (3.5 mm; Figure 1A). There were collaterals from his septal left anterior descending coronary artery to his distal right coronary artery (Figure 1B).

**Figure 2:**
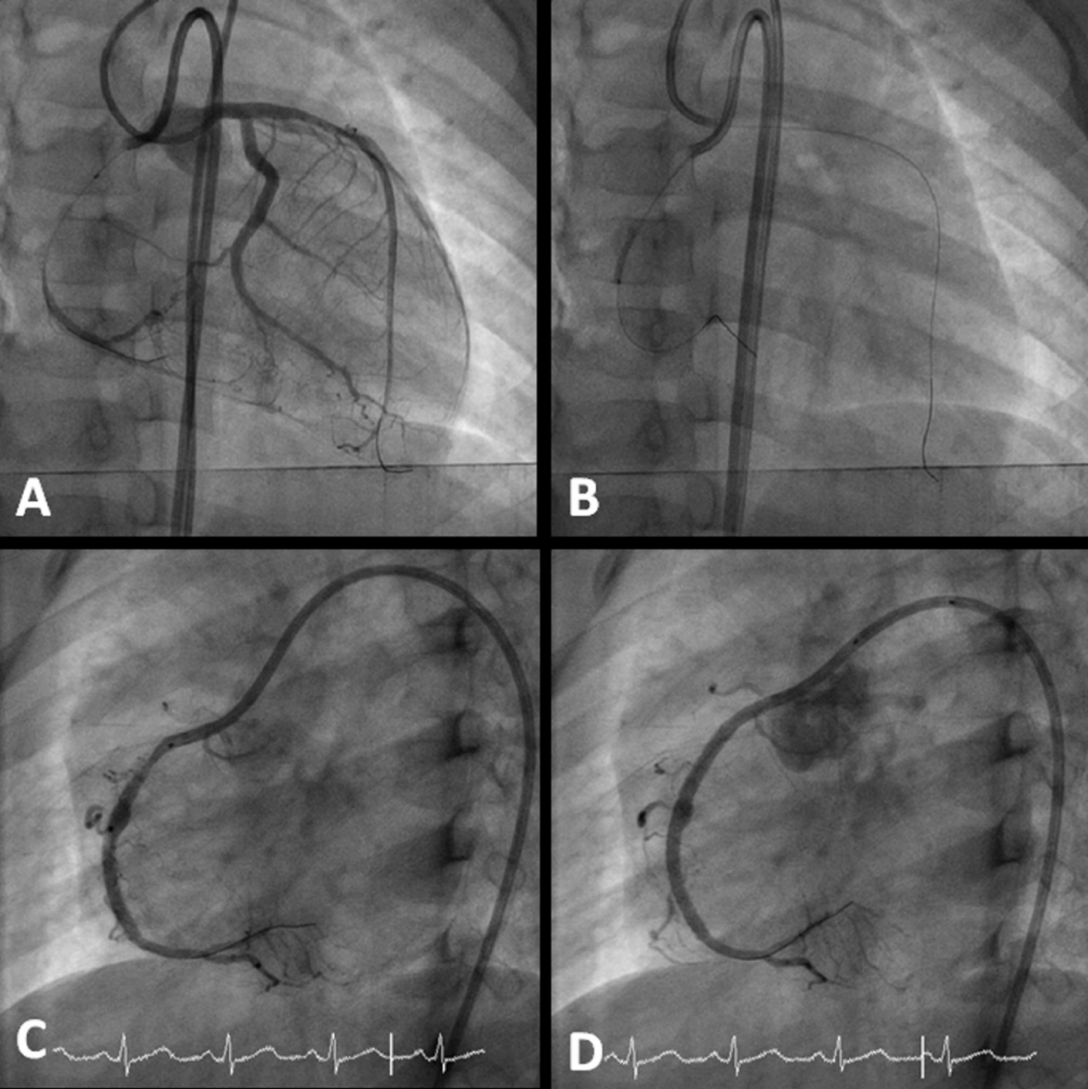
**Stent implantation**. **(A) **A 0.014-inch guidewire was advanced to cross the totally occluded site with a 1.5 mm balloon catheter back-up. **(B) **Pre-dilating the middle portion of the right coronary artery. **(C) **After deploying the first stent (2.5 × 28 mm). **(D) **After deploying the second stent (2.5 × 18 mm).

A Launcher Coronary Guiding Catheter (6Fr SCR 3.5 Medtronic, Danvers, MA, USA) was placed against the opening of his right coronary artery. Two holes were created on the sides of the guiding catheter to prevent pressure damping. A 0.014-inch Choice PT2 Light Support Guidewire (Boston Scientific, Miami, FL, USA) was then advanced across the totally occluded site with a 1.5 mm Sprinter Over-the-Wire Semicompliant Balloon Dilatation Catheter (Medtronic, Galway, Ireland) back-up (Figure 2A). After wire crossing, we confirmed distal true lumen entry by injecting contrast to the collateral arteries from his left anterior descending artery. After pre-dilating the middle portion of his right coronary artery with the 1.5 mm balloon up to 12 bar (Figure 2B), stenting of his right coronary artery was accomplished using a 2.5 × 28 mm and a 2.5 × 18 mm Mini Vision bare metal stent (Abbott Vascular, County Tipperary, Ireland; Figure 2C, D). A final angiography demonstrated no residual stenosis or dissection. The pulsation and perfusion were good after the procedure. Our patient received anti-platelet therapy with aspirin and clopidogrel for three months, followed by aspirin only thereafter.

## Discussion

Whether or not an asymptomatic young children should be treated for a totally occluded coronary artery after Kawasaki disease is a controversial issue [2,4-6]. According to recent guidelines for adult coronary heart disease, intervention for patients without evidence of ischemia is not recommended [7]. Similarly, earlier Japanese guidelines did not support intervention in such cases [6]. However, children with a coronary artery lesion after Kawasaki disease still have a long life expectancy, but living with those collateral arteries might carry an increased risk of myocardial infarction and dilated cardiomyopathy later in life [8]. In the largest series of coronary intervention after Kawasaki disease in Japan, the authors stated that patients with stenotic lesions of ≥ 75% were indicated for coronary intervention even without evidence of ischemia [9]. In fact, about one-third of patients receiving percutaneous coronary intervention and one-seventh of patients receiving coronary artery bypass grafting for Kawasaki disease sequelae were asymptomatic in Japan [4]. In our patient, a conservative treatment approach would have meant that he would have been depending on a single coronary artery for possibly 70 years or more. Furthermore, his left coronary artery also had a mild stenosis. We therefore felt that aggressive treatment would be a better approach for such a young boy.

For coronary sequelae after Kawasaki disease, it remains unclear which of the two main approaches, percutaneous coronary intervention or coronary artery bypass grafting, is superior. In a recent questionnaire survey in Japan that used death or Q-wave myocardial infarction as the primary endpoints there were no significant differences between these two methods. But, for the secondary endpoint of repeat revascularization, coronary artery bypass grafting had a lower re-intervention rate, especially for patients with ischemic change and in those younger than 12 years old [4]. However, in the subgroup analysis for asymptomatic patients, there was no significant difference. There are two possible reasons. First, strong competition existed between the graft and collateral arteries in asymptomatic patients [8]. Second, because of the abundance of collaterals, the need for re-intervention was underestimated. The authors concluded that coronary artery bypass grafting should be recommended in younger patients having ischemic changes with multivessel disease [4]. In our patient, we considered percutaneous coronary intervention to be a more acceptable modality for an asymptomatic young boy.

For children as young as six years old, stent implantation for coronary lesions after Kawasaki disease is seldom reported. In a report by Ishii *et al*., stents were placed only for children older than 14 years [9]. In a series reported by Muta and Ishii, only 23% (16 out of 67) patients underwent stent implantation. The mean age of intervention was 16 years old [4]. Waki and Baba reported stenting a coronary artery in an eight-year-old boy. However, the boy's body weight reached 36.9 kg [10].

The re-stenosis rate after simple balloon dilatation of the lesion is as high as 25% to 50% in Japan [5,9]. The high rate may be attributed to marked intimal thickening and calcification secondary to arteritis [2]. Simple balloon dilatation without stent implantation may result in another possible complication, formation of a new aneurysm. It may be caused by dissection of the vascular wall due to a high-pressure balloon [5,9]. Therefore, a maximal balloon pressure of less than 8 to 10 atm has been suggested. Stenting the site of the coronary artery stenosis or aneurysm might prevent the development of the two abovementioned phenomena. However, there are currently insufficient data to reach a definitive conclusion on this issue. If the lesion has existed for more than six years after the onset of symptoms, rotational ablation should be considered because of the high failure rate of balloon dilatation or stent implantation due to severe calcification [2]. In most reported patients, bare metal stents were used. The use of a covered stent has also been described [10]. Li *et al. *reported that an aneurysm was found to worsen after placing a drug-eluting stent [11], therefore drug-eluting stents should be used with caution. Akagi suggested that stenting should only be considered in children older than 12 years due to the large vascular access needed [2]. However, in this patient, a 6Fr guiding catheter (after creating side holes) could be used smoothly in a six-year-old boy.

The use of percutaneous coronary intervention for treatment of the sequelae of Kawasaki disease has limitations. First, for children under six years old, the vascular size may be too small to safely perform the procedure. Second, we have to take somatic growth into account when stent implantation is planned, especially in small children. Therefore, we have to think about whether an additional dilatation is possible or not, and eventually how much maximal diameter can be obtained. For children under 12 years old with ischemic changes and evidence of disease in multiple vessels, coronary artery bypass grafting may be a better choice [4].

## Conclusion

Stent implantation can be safely performed in children as young as six years old. Early diagnosis of a coronary lesion after Kawasaki disease and early treatment may obviate the need for a complicated procedure later in life. Close follow-up is mandatory because the long-term outcome is still unclear, especially in such a small child.

## Consent

Written informed consent was obtained from the patient's mother for publication of this case report and any accompanying images. A copy of the written consent is available for review by the Editor-in-Chief of this journal.

## Competing interests

The authors declare that they have no competing interests.

## Authors' contributions

YCH wrote the manuscript. MCL reviewed the article. KWL helped do the procedure. YCF and SLJ provided their experience in caring for patients of Kawasaki disease in the discussion section. All authors read and approved the final manuscript.

## References

[B1] McCrindle BW Li JS Minich LL Colan SD Atz AM Takahashi M Vetter VL Gersony WM Mitchell PD Newburger JW Coronary artery involvement in children with Kawasaki disease: risk factors from analysis of serial normalized measurements Circulation 2007 116 174 179 10.1161/CIRCULATIONAHA.107.690875 17576863

[B2] Akagi T Interventions in Kawasaki disease Pediatr Cardiol 2005 26 206 212 10.1007/s00246-004-0964-2 15868317

[B3] Lin MC Hsu CM Fu YC Giant coronary aneurysms developed in a child of Kawasaki disease with only 3 days of fever Cardiol Young 2010 20 339 341 10.1017/S104795111000017X 20307334

[B4] Muta H Ishii M Percutaneous coronary intervention versus coronary artery bypass grafting for stenotic lesions after Kawasaki disease J Pediatr 2010 157 120 126 10.1016/j.jpeds.2010.01.032 20304414

[B5] Akagi T Ogawa S Ino T Iwasa M Echigo S Kishida K Baba K Matsushima M Hamaoka K Tomita H Ishii M Kato H Catheter interventional treatment in Kawasaki disease: a report from the Japanese Pediatric Interventional Cardiology Investigation group J Pediatr 2000 137 181 186 10.1067/mpd.2000.107164 10931409

[B6] Ishii M Ueno T Akagi T Baba K Harada K Hamaoka K Kato H Tsuda E Uemura S Saji T Ogawa S Echigo S Yamaguchi T Kato H Research Committee of Ministry of Health, Labour and Welfare--"Study of treatment and long-term management in Kawasaki disease" Guidelines for catheter intervention in coronary artery lesion in Kawasaki disease Pediatr Int 2001 43 558 562 10.1046/j.1442-200X.2001.01464.x 11737728

[B7] King SB Smith SC Jr Hirshfeld JW Jr Jacobs AK Morrison DA Williams DO Feldman TE Kern MJ O'Neill WW Schaff HV Whitlow PL ACC/AHA/SCAI Adams CD Anderson JL Buller CE Creager MA Ettinger SM Halperin JL Hunt SA Krumholz HM Kushner FG Lytle BW Nishimura R Page RL Riegel B Tarkington LG Yancy CW 2007 focused update of the ACC/AHA/SCAI 2005 guideline update for percutaneous coronary intervention: a report of the American College of Cardiology/American Heart Association Task Force on Practice guidelines J Am Coll Cardiol 2008 51 172 209 10.1016/j.jacc.2007.10.002 18191745

[B8] Tsuda E Fujita H Yagihara T Yamada O Echigo S Kitamura S Competition between native flow and graft flow after coronary artery bypass grafting. Impact on indications for coronary artery bypass grafting for localized stenosis with giant aneurysms due to Kawasaki disease Pediatr Cardiol 2008 29 266 270 10.1007/s00246-007-9114-y 17917764

[B9] Ishii M Ueno T Ikeda H Iemura M Sugimura T Furui J Sugahara Y Muta H Akagi T Nomura Y Homma T Yokoi H Nobuyoshi M Matsuishi T Kato H Sequential follow-up results of catheter intervention for coronary artery lesions after Kawasaki disease: quantitative coronary artery angiography and intravascular ultrasound imaging study Circulation 2002 105 3004 3010 10.1161/01.CIR.0000019733.56553.D8 12081995

[B10] Waki K Baba K Transcatheter polytetrafluoroethylene-covered stent implantation in a giant coronary artery aneurysm of a child with Kawasaki disease--a potential novel treatment Catheter Cardiovasc Interv 2006 68 74 77 10.1002/ccd.20808 16763997

[B11] Li SS Cheng BC Lee SH Images in cardiovascular medicine. Giant coronary aneurysm formation after sirolimus-eluting stent implantation in Kawasaki disease Circulation 2005 112 e105 107 10.1161/CIRCULATIONAHA.104.503300 16116061

